# Estimation of pelvis kinematics in level walking based on a single inertial sensor positioned close to the sacrum: validation on healthy subjects with stereophotogrammetric system

**DOI:** 10.1186/1475-925X-13-146

**Published:** 2014-10-21

**Authors:** Francesca Buganè, Maria Grazia Benedetti, Valentina D’Angeli, Alberto Leardini

**Affiliations:** LetSense Srl, via Bruno Buozzi 25, Castel Maggiore, 40013 Italy; Physical Medicine and Rehabilitation Unit, Istituto Ortopedico Rizzoli, via G. C. Pupilli 1, Bologna, 40100 Italy; Movement Analysis Laboratory, Istituto Ortopedico Rizzoli, via di Barbiano 1/10, Bologna, 40100 Italy

**Keywords:** Pelvis kinematics, Walking, Gyroscope, Validation, Gait analysis, Inertial motion units

## Abstract

**Background:**

Kinematics measures from inertial sensors have a value in the clinical assessment of pathological gait, to track quantitatively the outcome of interventions and rehabilitation programs. To become a standard tool for clinicians, it is necessary to evaluate their capability to provide reliable and comprehensible information, possibly by comparing this with that provided by the traditional gait analysis. The aim of this study was to assess by state-of-the-art gait analysis the reliability of a single inertial device attached to the sacrum to measure pelvis kinematics during level walking.

**Methods:**

The output signals of the three-axis gyroscope were processed to estimate the spatial orientation of the pelvis in the sagittal (tilt angle), frontal (obliquity) and transverse (rotation) anatomical planes These estimated angles were compared with those provided by a 8 TV-cameras stereophotogrammetric system utilizing a standard experimental protocol, with four markers on the pelvis. This was observed in a group of sixteen healthy subjects while performing three repetitions of level walking along a 10 meter walkway at slow, normal and fast speeds. The determination coefficient, the scale factor and the bias of a linear regression model were calculated to represent the differences between the angular patterns from the two measurement systems. For the intra-subject variability, one volunteer was asked to repeat walking at normal speed 10 times.

**Results:**

A good match was observed for obliquity and rotation angles. For the tilt angle, the pattern and range of motion was similar, but a bias was observed, due to the different initial inclination angle in the sagittal plane of the inertial sensor with respect to the pelvis anatomical frame. A good intra-subject consistency has also been shown by the small variability of the pelvic angles as estimated by the new system, confirmed by very small values of standard deviation for all three angles.

**Conclusions:**

These results suggest that this inertial device is a reliable alternative to stereophotogrammetric systems for pelvis kinematics measurements, in addition to being easier to use and cheaper. The device can provide to the patient and to the examiner reliable feedback in real-time during routine clinical tests.

## Background

Gait is the manner in which human walking is performed [1], and the ability to walk upright is its main defining characteristic [2]. This task requires the coordinated control of nearly every segment of the neuromusculoskeletal system, but pelvis motion plays a fundamental role. This motion is referred to also as one of the six main mechanical contributions to flatten the patterns of vertical and horizontal displacement of the body’s center of mass [3], and therefore pelvis kinematics reveals much of energy expenditure. This is usually described in a laboratory reference frame, mainly in terms of angle time-histories in the three anatomical planes. During normal gait, typical patterns of these rotations are expected. In particular the pelvis drops 4–5 degrees away from the stance leg and toward the swing leg also to decrease the necessary displacement of the center of mass in the transverses plane during single limb support, this phenomenon being referred to also as Trendelenburg [4]. Pelvic rotations are also responsible for the main spatio-temporal parameters of gait, i.e. step and stride lengths, velocity, cadence etc. The importance of pelvic movement in the elderly was pointed out a long ago [5]. The patterns of this movement during walking are used to identify instability, which is the main risk factor associated with fall in this population. The pelvis also shows the basis of many gait abnormalities, such as anterior pelvic tilt, contralateral pelvic drop and ipsilateral pelvic drop. These abnormalities frequently result in muscle imbalance and relevant compensation mechanisms in other segments. In patients with cerebral palsy, for example, the characteristic irregular pattern of pelvic retraction (external rotation) is commonly observed, as a compensation for the increased internal hip rotation [6]. Pelvic motion by standard gait analysis has been described largely also for amputees, particularly by examining possible normative patterns which may help to explain important aspects of their walking and provide insight as to how to improve prosthetic gait [7–9].

Gait analysis is the systematic measurement, description and assessment of those quantities thought to characterize human locomotion. Using stereophotogrammetric systems and force platforms, kinematic and kinetic data are acquired and analyzed for a number of segments and joints, ultimately interpreted by the clinicians to form an assessment. Pelvic kinematics is usually analyzed in these gait analysis laboratories through a number of different marker sets, but more recently it has been analyzed also by inertial sensors [10]. A most traditional technique involves four markers placed at the anterior and posterior superior iliac spines (two ASISs and two PSISs) [11, 12], though technical clusters of markers fixed on a rigid base attached to the lateral side or to the sacrum have also been used [13, 14]. The use of wand markers on the pelvis to reconstruct virtually the ASIS markers has been proposed recently [15] to overcome visibility problems in overweight subjects. Several studies however have pointed out the inaccuracies associated with the relative movement between surface markers and underlying bones [16] and the pelvic soft tissue artifacts in multiple static calibration positions [17]. Nonetheless, pelvic kinematics is still the most reliable measure in clinical standard gait analysis with the lowest errors in pelvic rotation and obliquity [18].

Unfortunately, these standard gait analysis laboratories require large rooms, expensive equipment, specialized personnel, lengthy set-ups and intricate post processing calculations. To overcome these problems the utilization of wearable inertial sensors have been proposed, particularly because much less expensive, smaller and usable also outside [19]. Several relevant applications in natural walking characterization [20–22] and in patient rehabilitation have been reported already [23–25]. To obtain more information on human gait, other wearable sensors, such as gyroscopes, have been combined with accelerometers in the so-called Inertial Measurement Units. Different techniques have been proposed also to calculate position and orientation in space of these devices by different filters, such as the complementary or the Kalman [21, 23] or the weighted Fourier linear combiner [22]. This latter approach has been applied successfully to patients with stroke and Parkinson’s diseases [24]. In all these studies only lower trunk angles were analyzed, and the results obtained were compared with data provided by a stereophotogrammetric system by simply placing markers directly on the device, not on the skin as in the traditional gait analysis.

Despite wide clinical interest, the use of inertial sensors to estimate specifically pelvis kinematics has rarely been addressed. Acceleration patterns at the head and pelvis has been evaluated [26] while subjects walked on a level and on an irregular walking surface, with the purpose of understanding how the postural control system responds to challenging walking conditions. Only linear acceleration of the body was measured, however, along three orthogonal axes using two triaxial accelerometers, one at the head and one affixed to a plate firmly strapped onto the subject with a belt at the level of the sacrum. Only temporal and spatial gait parameters and acceleration patterns at the head and pelvis were measured, with no interest in pelvis angles. More recently, the pelvic movement has been analyzed in the elderly [27] during walking using a wireless posture monitoring system equipped with a triaxial accelerometer and a triaxial gyroscope. The lower end of the sensor was placed on the center of the second sacral spine and rigidly fixed by an elastic band wrapped around the pelvis. This system was able to measure angular positions, velocities and accelerations in the three axial directions. Pelvic kinematics during walking was compared between two groups of elderly subjects. In Bolink et al. [28] a single inertial sensor located at the pelvis was used to derive various motion parameters also during walking. They compared preoperative measures of the end-stage knee osteoarthritis patients to healthy subjects by means of calculation of spatiotemporal gait parameters and lumbo-pelvic ranges of motion. However in both these papers there is no reference to the gold standard of stereophotogrammetry.

There is therefore ample new literature on the estimation of human motion and spatio-temporal parameters using inertial sensors, but rarely for pelvis kinematics, and a few less have validated these systems by state-of-the-art gait analysis (GA) via stereophotogrammetry. Pelvis kinematics measurements by a single sensor would be very useful in the clinical assessment of pathological gait, particularly in the context of rehabilitation, to assess quantitatively the outcome of specific interventions on gait recovery. Such systems can also support the orthopedist to verify the correct alignment of the pelvis in amputees and in any lower limb intervention, currently assessed only through clinical observations. This would be possible with the minimum encumbrance for the patient and very low costs for the health-care service. However, to make inertial devices readily accessible and reliable, it is necessary to assess their capability in-vivo to offer information comparable with those provided by the gold standard of GA [25, 29], which has provided the traditional corresponding measures for decades. The present study was aimed at investigating whether a single inertial sensor on the sacrum can provide reliable pelvis kinematics in level walking. This must be assessed experimentally, in a number of different subjects, and possibly at a number of walking velocities because the inertial-based measurements can be affected by many sources of artifacts, such as the shape and positioning of the sensor, soft tissues deformation and motion, sensor vibration, etc. This assessment was initially performed only in a population of healthy subjects, as a necessary first step before similar analyses in a series of pathological populations.

## Methods

The specific scope of the present study was to assess the performance of a technique based on a wireless inertial sensing device, Free4Act (F4A – LetSense Srl, Bologna, Italy) to estimate human pelvis angles during walking. The results obtained with the F4A sensor were compared with corresponding ones obtained by the gold standard of GA.

The system and the method used to perform the measurements and to validate the results are the following.

The data here analyzed were collected within a series of non-invasive measures routinely taken in the movement analysis laboratory; according to the ethical policies of the institute, informed consent was obtained from the volunteers, after careful explanation of the scopes, the instruments and the techniques used for thepresent investigation.

### The inertial sensing and gait analysis systems

**Figure 1:**
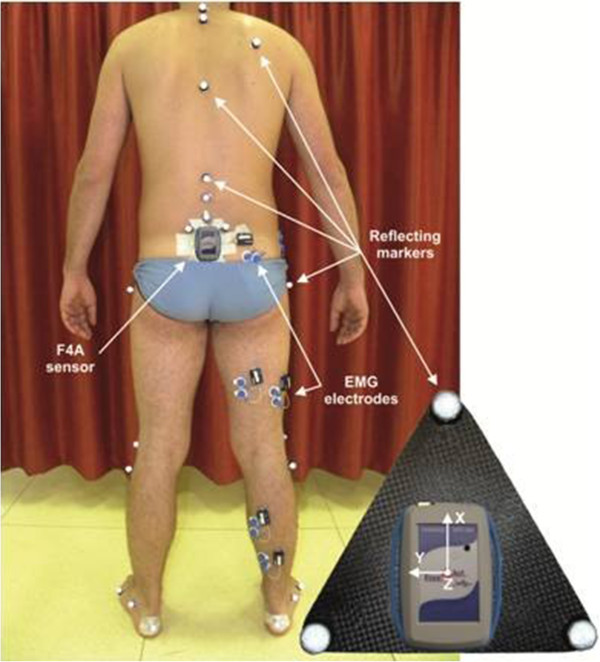
**The marker set and F4A sensor positions on the sacrum in a representative subject.** Image taken from the experimental session. Electrodes for EMG signal data collection can also be seen, though not addressed in the present study. The inset shows the triangular rigid plate with the three marker cluster and the F4A with its reference frame bonded to each other.

The inertial system used was the same described in a previous work by the present authors [20], but in the present study both the triaxial accelerometer and the gyroscope were used. The F4A sensor (Figure 1) was attached to the subject’s sacrum determined by palpation and using adhesive plasters, in a way that accelerations and angular velocities could be collected about the three orthogonal anatomical axes of the pelvis, i.e. the anterior-posterior, medial-lateral and cranio-caudal. The procedure used in the present study for F4A positioning recommends that the upper side of the cage be aligned with the axis between the two markers on the PSISs. From the collected gyroscope signals, the pelvic angles on the three anatomical planes were obtained, which are tilt (sagittal plane), obliquity (frontal plane) and rotation (transverse plane). From the collected acceleration signals the static angles in the sagittal and frontal planes, which are the inclination angles of the F4A sensor when the subject was standing still in an up-right double-leg posture, were also calculated using it as an inclinometer. Later these static angles were added to the pelvic angles obtained by the gyroscope signals to express F4A data in the laboratory reference frame.

The validation of the overall F4A inertial system, made of all instrumentation including the sensor, plus all relevant algorithms and software, was performed by using a GA system, which included a stereophotogrammetric unit with eight M2-cameras (Vicon 612, Vicon Motion Capture, Oxford, UK) and two dynamometric platforms (Kistler Instruments, Einterthur, Switzerland), sampling respectively at 100 and 1000 Hz. For marker positioning and lower limb 3-D kinematics, a recently established protocol for clinical analysis was used [12]. This involves fixing 35 markers on the skin in relevant anatomical locations, 6 of which are removed after a calibration procedure based on a static posture acquisition (Figure 1). Based on these markers, trunk and pelvis three-dimensional (3D) orientations in the laboratory reference frame are calculated, as well as the three-dimensional angles and moments of the lower limb joints during the execution of the motor task under analysis.

### Experimental setting

Sixteen healthy young individuals (7 men and 9 women, age range 19–39 years, average age 26 ± 3 years; height range 156–190 cm, average height 171 ± 9 cm; mass range 48–106 kg, average mass 64 ± 16 kg, BMI range 29–16, average BMI 21 ± 3), participated in the study after giving their informed consent. The subjects were recruited among students of the University of Bologna and none had a previous history of musculoskeletal, neurological or other gait disorders.

Each subject was instrumented with the relevant reflective markers and the F4A sensor (Figure 1). This was placed on the sacrum so that anterior-posterior, medial-lateral and vertical directions coincided with those of the pelvis, respectively z-axis, y-axis and x-axis. The sensitivity for the F4A accelerometer of ±1.5G and for the gyroscope of ±300°/s was chosen. Data of the accelerometer and the gyroscope was sampled at 100 Hz. The subjects were asked to stand up and remain in the up-right posture for a few seconds, and then asked to walk barefoot along a 10-m pathway at three different speeds, slow, self-selected and fast. This entailed overall 8–12 steps, i.e. progression of one leg, according to the subject’s natural cadence and height; the central three steps, i.e. one left and right full gait cycle, were analyzed also by the GA system. This exercise was repeated 3 times, hereinafter trials, for each participant at each different walking speeds.

In five subjects, two trials at self-determined speed with the F4A sensor rigidly bonded to a triangular plate mounting a cluster of three markers were also acquired (Figure 1, right-bottom inset), to verify directly the correct tracking of the motion data provided by the F4A system. For these additional trials, the same triangle vertex was placed instead of the marker on the five lumbar vertebrae (L5) (Figure 1).

For the intra-subject variability, one volunteer was asked to repeat the level walking at normal speed 10 times.

### Data processing

Relevant raw data both from the accelerometer and gyroscope units were first filtered by a fourth-order Butterworth low-pass filter with a cut off frequency of 8 Hz [30]. To calculate the inclination angles of the F4A sensor in the static posture, the three acceleration signals, stored for a few seconds when the subject stood up and remained in the up-right posture before starting walking, were elaborated, using the accelerometer as a sort of inclinometer. The three pelvic angles were estimated by integrating the three gyroscopic signals plus the subsequent removal of the drift using the function ‘detrend’ in Matlab (Mathworks, Natick MA USA) which removes the best straight-line fit linear trend from a signal. For both F4A- and GA-based angle pattern calculations, the gait cycle, i.e. from heel-strike to heel-strike of the same foot, was identified for each side, as in previously reported studies [12, 20]. The time-history of three pelvis angles were then normalized in time by re-sampling these values over 100 samples; in this way the axis of abscissae reports the percentage of gait cycle. For the latter, the first heel strike event was taken from the corresponding force platform and the following corresponding event from the comparison of ankle joint kinematics. Pelvic kinematics was calculated for both the right and left leg cycles, assuming GA-based 3D calculations as gold-standard reference angles. For this comparison, the estimated three angles from F4A during gait were corrected by adding to the corresponding initial static angle for the orientation of the F4A (offset angles). Finally, for the special trials, 3D orientation of the triangular plate was also reconstructed using the stereophotogrammetric data of its markers; a technical reference frame was defined embedded on these three markers, and the orientation of this in the laboratory frame was calculated.

The comparison between the F4A and GA system measurements was performed via correlation of the corresponding patterns of motion, as well as for the range of this motion, i.e. maximum and minimum value difference. The correlation was performed on data from the second trial, between the average pattern over the steps collected by the F4A system, and the corresponding pattern from GA in collected single cycle; this was replicated for each angle, each progression speed and each subject.

### Statistical analysis

The reliability of data provided by F4A system was first verified by comparing them to the angular values obtained from the three marker positions of the triangular plate during the special trials, at self-selected speed. The root mean square difference (RMS), the correlation coefficient (r) and the differences between the mean values of corresponding angles curves were computed for this purpose [24].

To assess the performance of the F4A system to track the pelvis, the angle estimates were compared also with the reference values from the GA protocol [12]. The determination coefficient (r^2^), the scale factor (a) and the bias (b) of a linear regression model were adopted to compare the angular patterns from the two measurement systems [31, 32]. For the present statistical analysis, only the second trial for each walking speed was analyzed, to allow the subject to get familiar with the motor task and to avoid possible alterations at the last trials. Furthermore the estimated angular ranges were compared with those obtained from standard GA using the paired *t*-test, after checking the assumptions of Gaussian distribution of the data and the correlation between the compared measures. For the *t*-test, significance was assumed for p-values smaller than 0.05. Statistical analysis was performed by Statistical Package for the Social Sciences (SPSS) software version 15.0 (SPSS Inc, Chicago, IL).

## Results

### Technical comparison by triangular plate

**Figure 2:**
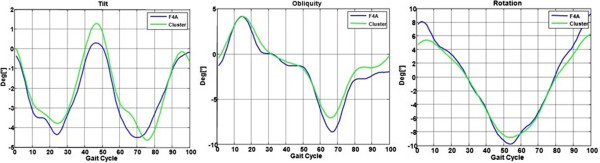
**Technical comparison by triangular plate.** Superimposition of the estimated angles provided by the F4A (blue lines) and the corresponding reference angles calculated with the markers on the associated triangular plate (green lines), as recorded during one typical trial of a randomly selected subject, for each of the three anatomical axes (three plots). This technical comparison was made in the reference frame of the three marker cluster.

The angles from F4A and those from relevant triangular plate orientation calculation from stereophotogrammetry were found to be very similar in all five subjects, both in their patterns and in their ranges (Figure 2). This observation is also supported by RMS and r values (Table 1): RMS was smaller than 1° for tilt angle and smaller than 3° for the obliquity and rotation angles. An average correlation coefficient r of approximately 0.90 was obtained for all three angles, the highest value being observed for obliquity, 0.95. The average distance was smallest for tilt, about 0.1° and largest for rotation, about 1.8°.

**Table 1:** **Difference between the estimated angles provided by the F4A and the triangular plate**

Angles	RMS	r	Difference
Tilt	0.73 (0.38)	0.88 (0.04)	0.12 (0.13)
Obliquity	1.22 (0.41)	0.95 (0.02)	0.85 (0.46)
Rotation	2.66 (1.57)	0.91 (0.11)	1.76 (1.03)

Mean and standard deviation of the difference between the estimated angles provided by the F4A and the relevant plate orientation angles, as obtained over 5 representative subjects. Unit is degree.

### F4A versus GA: intra-subject repeatability

**Figure 3:**
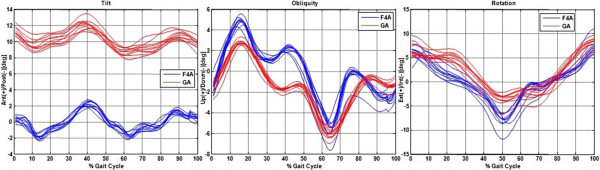
**F4A versus GA: intra-subject repeatability.** Pelvic angles about the three axes provided by F4A sensor (blue lines) as the mean of all the gait cycles detected, and by the GA system (red lines), expressed in the laboratory reference frame; for both, 10 repetitions of the right leg are superimposed, for the same representative subject. Just for this comparison, static angles were not added to F4A data.

A good intra-subject consistency of the pelvic angles, both from F4A and GA, was also observed, as appreciable by a superimposition of relevant curves (Figure 3). Within the two systems, the standard deviations of these 10 curves when normalized over the 100 samples of the gait cycle were smaller than 0.3, 0.6 and 1.1 for tilt, obliquity and rotation angles respectively.

### F4A versus GA: overall comparison

**Figure 4:**
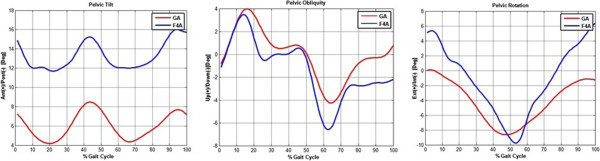
**F4A versus GA: patterns comparison.** Pelvic angles about the three axes, as provided by F4A as the mean of all the detected cycles (blue lines), and those provided by GA in the second trial (red lines), expressed in the laboratory reference frame; patterns from a single representative subject at self-selected speed are shown.

The pelvic angles from F4A also compare well with those calculated by the anatomical GA protocol, but only for obliquity and rotation (Figure 4). For tilt, i.e. pelvic angles in the sagittal plane, the patterns over the gait cycle are very similar, both forward as known in human kinesiology, but a bias of about 7° - 8° is evident: F4A angles range between 12° and 16°, GA angles between 4° and 8°. This bias is due to the different inclination angles in the sagittal plane measured by the two systems during the subject’s static position, about 9° (Table 2). This is further supported by the fact that tilt from F4A, 21.2°, nearly corresponds to the plate on the sacrum, 22.2°. On the other hand, obliquity of the pelvis is very well represented by the F4A in the static up-right posture (Table 2).

**Table 2:** **Comparison of static inclination angles provided by F4A, by GA and by the tringular plate**

Static Angles	F4A	GA	Cluster
Tilt	21.23 (2.28)	11.69 (2.49)	22.24 (2.17)
Obliquity	-1.22 (3.40)	0.31 (1.28)	-1.87 (2.62)

The static inclination angles in the sagittal (tilt) and frontal (obliquity) planes as estimated by the F4A, as calculated by GA, and by the triangular plate of markers: mean and standard deviation over 5 representative subjects. Rotation angle cannot be calculated by the F4A in static because it is worked out by the inclinometer in only two anatomical planes. Unit is degree.

A good correlation between F4A- and GA-based time series of the angles was found in obliquity and rotation, poor for tilt, irrespectively of the walking speed (Tables 3, 4 and 5). For the purpose of a better representation, the values of the determination coefficient were divided into three intervals (0.0 - 0.6, 0.6 - 0.8 and 0.8 – 1.0) in each walking speed (slow: 1.01 ± 0.1 m/s; normal: 1.34 ± 0.7 m/s; fast: 1.56 ± 0.14 m/s). At self-selected speed, a high determination coefficient of approximately 0.9 was obtained for obliquity and rotation angles in most of the subjects (10 and 11 respectively, out of 16), thus showing a high angle pattern similarity (Tables 3 and 4). For these two angles, ranges of motion (somehow represented by the scale factor a, Table 5) compare well (between 0.9 and 1.3, being no scaling =1), and also the bias (b) is very small (smaller than 1.5°). For the tilt, less satisfactory results were obtained, revealed by the large number of subjects in the low determination coefficient interval at each walking speed (Table 3). However, for this angle the GA range of motion was well represented by the F4A in normal walking (a = 0.94), whereas a considerable bias (b) was introduced (Table 5), as discussed already here above.

**Table 3:** **Number of subject for each of three determination coefficient intervals and for each walking speed**

n° Subjects	Self-Selected			Slow			Fast		
	0-0.6	0.6-0.8	0.8-1	0-0.6	0.6-0.8	0.8-1	0-0.6	0.6-0.8	0.8-1
Tilt	12	2	2	7	6	3	12	2	2
Obliquity	2	4	10	2	4	10	2	3	11
Rotation	3	2	11	3	2	11	1	5	10

Number of subjects for each of the three determination coefficient (r^2^) intervals (0.0-0.6, 0.6-0.8 and 0.8-1.0), and for each walking speed; the three angles in the three rows.

**Table 4:** **Mean and standard deviation of the determination coefficient for each walking speed**

r ^2^	Self-Selected			Slow			Fast		
	0-0.6	0.6-0.8	0.8-1	0-0.6	0.6-0.8	0.8-1	0-0.6	0.6-0.8	0.8-1
Tilt	0.31(0.19)	0.68(0.09)	0.85(0.01)	0.33(0.16)	0.72(0.04)	0.85(0.03)	0.23(0.21)	0.62(0.03)	0.87(0.06)
Obliquity	0.46(0.12)	0.66(0.08)	0.88(0.06)	0.30(0.20)	0.75(0.03)	0.89(0.05)	0.35(0.05)	0.69(0.07)	0.89(0.04)
Rotation	0.25(0.19)	0.66(0.01)	0.91(0.06)	0.35(0.22)	0.71(0.09)	0.93(0.05)	0.36(0.20)	0.76	0.91(0.06)

Mean and standard deviation of the determination coefficient (r^2^) divided into the three intervals (0.0-0.6, 0.6-0.8 and 0.8-1.0) for each walking speed; the three angles in the three rows. Unit is degree.

**Table 5:** **Scale factor and bias of a linear regression model for the time-history of the angles**

Angles	Self-Selected		Slow		Fast	
	a	b	a	b	a	b
Tilt	0.94(0.45)	-9.66(9.49)	0.86(0.78)	-9.88(10.1)	0.60(0.77)	-5.66(8.42)
Obliquity	1.25(0.40)	-1.24(1.47)	1.14(0.20)	-1.26(1.66)	1.32(0.26)	-1.41(2.27)
Rotation	1.19(0.52)	1.30(2.04)	0.91(0.58)	1.50(2.86)	1.16(0.48)	0.71(2.09)

The scale factor (a) and the bias (b) of a linear regression model for the time-history of the pelvic angles: mean and standard deviation over all subjects, for each walking speed; the three angles in the three rows. Unit is degree.

**Figure 5:**
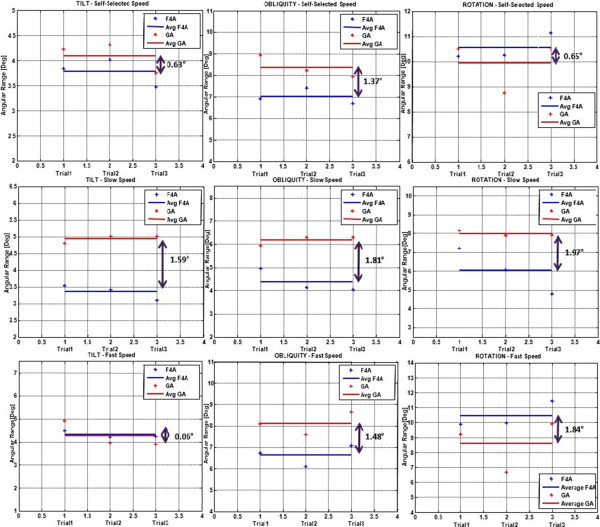
**F4A versus GA: angular ranges comparison.** Direct estimated angular ranges from F4A (blue crosses) and GA (red crosses) separately for each of the three collected trials, in one representative subject. The average over the three is superimposed (horizontal line segment; blue and red for F4A and GA). This plot is replicated for each of the three angles (columns) and each of the three walking speeds (rows).

A direct assessment of the estimated angular ranges from these two systems strengthens the conclusion that these angular measurements from the F4A represent well real pelvic motion (Figure 5). For this range of movement, the second trial only (Trial2) was also analyzed over all subjects (Table 6). In addition to the high repeatability of this inter-subject measurement, i.e. small standard deviations, the difference of the means from the two systems was smaller than 3° for each angle and each of the three different walking speeds. In only two of these nine comparisons the difference was statistically significant; no significant differences were found at the self-selected speed, the differences being found only in rotation at slow speed and in obliquity at fast speed (Table 6).

**Table 6:** **Comparison of mean and standard deviation of angular ranges as provided by F4A and GA**

Range	Self-selected			Slow			Fast		
	F4A	GA	***p***-Values	F4A	GA	***p***-Values	F4A	GA	***p***-Values
Tilt	3.52(1.08)	3.26(1.14)	0.25	3.08(1.18)	2.88(1.19)	0.38	4.78(2.10)	3.90(0.89)	0.09
Obliquity	8.50(2.67)	8.48(3.25)	0.91	6.50(3.43)	6.82(3.16)	0.31	10.21(3.46)	9.49(3.66)	0.03*
Rotation	11.70(5.17)	11.78(5.15)	0.83	7.55(3.36)	10.13(3.35)	<0.001*	15.10(6.53)	14.48(5.31)	0.27

Range of motion as estimated by the F4A, and calculated by GA: mean and standard deviation over all subjects but for the second trial only (Trial 2 in Figure 5), together with corresponding p-values from the *t*-test: those significant are marked with *. This is reported for each of the three walking speeds, and each of the three angles. Unit is degree.

## Discussion

The aim of this study was to test experimentally in real conditions whether a novel wireless inertial system, made of the known sensor F4A [20] plus original algorithms and software, can provide reliably the time-histories of the three pelvis angles during level walking. For this purpose, walking at three different speeds, i.e. slow, self-selected and fast, was analyzed in a group of healthy young subjects by the F4A sensor on the sacrum. Pelvic angles estimated by this new system, based on all possible detectable gait cycles, were then compared to corresponding reference angles provided by a complete state-of-the-art GA system for the purposes of validation: this latter system in fact is nowadays the gold standard in the clinical settings for these measurements. Tilt, obliquity and rotation angles of the pelvis were calculated by integration of angular velocities as obtained from three orthogonally mounted gyroscopes inside the F4A sensor. To verify also the reliability of data provided by the F4A system in real conditions, these angles were compared with those calculated from the positions of the three markers placed at the same distance on a triangular plate hosting the F4A sensor rigidly, during two trials at self-selected speed for five subjects. This test confirmed the accuracy of the data provided by the F4A. The time-history of the angles provided by this new system also showed its good ability to track pelvis angular motion, as required in many clinical contexts. The present encouraging results compare well to what had been previously achieved in the monitoring of young healthy subject using more complex algorithms such as Kalman [21] or Weighted Fourier Linear Combiner adaptive filters [22, 24]. A good intra-subject consistency has also been shown by the small variability of the pelvic angles as estimated by the new F4A system, confirmed by the very small values of standard deviation for all three angles.

The patterns of the pelvis angles over the gait cycle here estimated by the F4A system also compare qualitatively well with corresponding normative angles reported in the literature from large populations of healthy subjects and by a variety of comprehensive instrumentations [11–13]. Only the pelvic tilt angle curves provided by F4A do not match well the known and measured motion of the pelvis in the sagittal plane, although similar trends are observed. However, this is associated to the different initial inclination angle, revealed by the results here reported in static up-right posture also by tracking the F4A device directly by three markers on the same plate. The anatomical justification can be identified simply in the different inclination of the sacrum with respect to the pelvis when observed traditionally via its ASIS and PSIS markers. Furthermore, the interposition of soft tissue between the F4A sensor and the sacrum can condition the inclination angle mainly in the sagittal plane. Finally, this measure might have been affected by fluctuations of the sensor due to unwanted rolling movement associated to the rounded shape of the case and its not completely stable fastening to the sacrum. These problems can be considerably reduced by the use of elastic bands for a more rigid attachment of the F4A sensor and more suitable designs of the case.

The present study is an initial part of a larger investigation, where normal subjects only are analyzed. Reasonably, every consistent pathological population will have own complications for the estimation of pelvis rotations by using this F4A system, particularly with respect to postural pelvis inclination in posture and gait. In the present initial study, it is assumed that the pelvis is in a general well aligned anatomical position. However, it is also expected that the present algorithms apply well on all those clinical populations where such pelvis condition is nearly well aligned. The observed offset in tilt likely involves the contribution, i.e. the sum, of the natural inclination of the pelvis and of the anatomical area when the F4A sensor is placed, but also that associated to possible unphysiological upright postures. For inter-subject comparisons, the offset removal is fundamental for both these sources of incorrect inclination. When intra-subject comparisons will be performed (for example in pre- versus post- treatment analyses, at various follow-ups, etc.) also the absolute rotation angles are definitely of interest; this are not reported here, but are calculated and stored by the present inertial sensing system. Moreover the range of motion of the pelvic rotations in each of the three anatomical planes is not large during level walking in healthy subjects, but can increase considerably in case of pathology or trauma of the lower limbs. Indeed, the good repeatability and consistency of the present measurements seem sufficient to get clear evidences and take important conclusions in clinical studies, as it has been shown already in the literature [6–8, 26–28].

A previous study by the present authors [20] showed the ability of the present wearable device, F4A, to estimate originally and reliably all relevant spatial-temporal parameters during level walking once mounted on the pelvis. The results obtained in the present study now suggest that this F4A sensor is a reliable alternative to stereophotogrammetric systems also for the evaluation of the angles in space for this segment. The simplicity of the overall apparatus and of the relevant experimental procedures, in addition to the simple signal processing algorithm here utilized, makes the F4A suitable for a large spectrum of clinical applications, even providing the physician and the patient with real-time feedback of pelvic motion. In the literature there are few previous studies reporting an analysis of the pelvis kinematics by a single inertial sensor placed on the sacrum [26–28], and the present work is in any case the first where estimated pelvic angles in level walking provided by an inertial sensor are compared with those traditionally provided by standard GA.

The potentialities and suitability of this new technique in routine clinical assessments, worked out by pointing out the differences between its measurements and those from a traditional GA system, have been identified. Range of motion is well represented by the F4A for each pelvic angle and each walking speed. Significant differences between the F4A and the GA system were found only in rotation at slow speed and in obliquity at fast speed. The first result was probably due to the decrease of the pelvis range of motion as a result of the decrease in walking speed, which makes the measurement harder. Indeed the rotation angle provided by the F4A is underestimated compared to the GA system. On the other hand, the increase in walking speed causes an increase of the device fluctuations, due to the already mentioned problems of its not completely stable fastening to the sacrum. In fact, the obliquity angle provided by the F4A is overestimated compared to the GA system, most likely due to unwanted movements emphasized by the higher speed. Nevertheless for both speeds a significant difference was detected in only one of the three angles investigated. A standard complete patterns of angular motion are replicated only for obliquity and rotation angles, i.e. in the frontal and transverse planes. A careful sensor mounting and an additional static acquisition are necessary to obtain reliable results. Inter-session repeatability should be investigated in future studies. Nevertheless, these issues do not diminish the overall value of the present novel instrumentation and technique; angular motion of the pelvis was here obtained reliably also with much simpler and cheaper technology than standard GA. In fact, the use of a single sensor reduces the costs of instrumentation and personnel, and the complexity of the data collection and analysis. This would allow such measurements to be taken routinely also in standard clinical settings, as well as those of sport and exercise, population aging, etc. for a more practical and economic monitoring tool of the functional performance of subjects, typically before and after treatments or rehabilitation programs, but also for the design and evaluation of prosthetics and orthotics. The present system provides automatically this relevant kinematics information, as well as other walking performance parameters such as the spatial-temporal data, as shown by the present authors in a previous paper [20].

Marked progress is expected in the future for this novel F4A-based system. As for the practical and experimental phase of the study, more reliable fixations of this sensor to the pelvis shall be investigated, by looking at specific elastic bands or double-sided stickers. In the near future improvements in the present validation work might be made by extending the present sampled population size, possibly looking at subjects grouped by gender, age, BMI, etc., and also at a number of pathological populations. Other motor tasks can also be investigated, such as those typically collected in clinical gait analysis, such as stair climbing/descending, chair raising/sitting, squatting, etc. Eventually, the quality of level walking before and after relevant treatments, as well as rehabilitation programs can be monitored with these measurements in a cheap and easy way.

## Conclusions

The results here obtained suggest that this inertial device is a reliable alternative to stereophotogrammetric systems for pelvis kinematics measurements, in addition to being easier to use and cheaper. The device can provide to the patient and to the examiner reliable feedback in real-time during routine clinical tests. The automatic and easy calculation of important spatial-temporal parameters [20] together with information about the pelvis kinematics make the F4A system a clinical tool which allows a complete gait analysis without challenging clinical interpretation and expensive instrumentations.

## References

[1] Malanga G, De Lisa JA, De Lisa JA (1998). Clinical observation. Gait Analysis in the Science of Rehabilitation. Section I.

[2] Iosa M, Fusco A, Moroni G, Paolucci S (2014). Development and decline of upright gait stability. Front Aging Neurosci.

[3] Saunders JB, Inman VT, Eberhart HD (1953). The major determinants in normal and pathological gait. J Bone Joint Surg.

[4] Huntley JS (2003). Trendelenburg and not Trendelenberg. Lancet.

[5] Murray MP, Kory RC, Clarkson BH (1969). Walking patterns in healthy old men. J Gerontol.

[6] Gaston MS, Rutz E, Dreher T, Brunner R (2011). Transverse plane rotation of the foot and transverse hip and pelvic kinematics in diplegic cerebral palsy. Gait Posture.

[7] Goujon-Pillet H, Sapin E, Fodé P, Lavaste F (2008). Three-dimensional motions of trunk and pelvis during transfemoral amputee gait. Arch Phys Med Rehabil.

[8] Tranberg R, Zugner R, Karrholm J (2011). Improvements in hip- and pelvic motion for patients with osseointegrated trans-femoral prostheses. Gait Posture.

[9] Rusaw D, Ramstrand N (2011). Motion-analysis studies of transtibial prosthesis users: a systematic review. Prosthet Orthot Int.

[10] Tao W, Liu T, Zheng R, Feng H (2012). Gait analysis using wearable sensors. Sensors.

[11] Kadaba MP, Ramakrishnan HK, Wootten ME (1990). Measurement of lower extremity kinematics during level walking. J Orthop Res.

[12] Leardini A, Sawacha Z, Paolini G, Ingrosso S, Nativo R, Benedetti MG (2007). A new anatomically based protocol for gait analysis in children. Gait Posture.

[13] Benedetti MG, Catani F, Leardini A, Pignotti E, Giannini S (1998). Data management in gait analysis for clinical applications. Clin Biomech.

[14] Borhani M, McGregor AH, Bull AM (2013). An alternative technical marker set for the pelvis is more repeatable than the standard pelvic marker set. Gait Posture.

[15] Smith M, Curtis D, Bencke J, Stebbins J (2013). Use of wand markers on the pelvis in three dimensional gait analysis. Gait Posture.

[16] Cappozzo A, Catani F, Leardini A, Benedetti MG, Della Croce U (1996). Position and orientation in space of bones during movement: experimental artifacts. Clin Biomech.

[17] Hara R, Sangeux M, Baker R, McGinley J (2014). Quantification of pelvic soft tissue artifact in multiple static positions. Gait Posture.

[18] McGinley JL, Baker R, Wolfe R, Morris ME (2009). The reliability of three-dimensional kinematic gait measurements: a systematic review. Gait Posture.

[19] Muro-de-la-Herran A, Garcia-Zapirain B, Mendez-Zorrilla A (2014). Gait analysis methods: an overview of wearable and Non-wearable systems, highlighting clinical applications. Sensors.

[20] Bugané F, Benedetti MG, Casadio G, Attala S, Biagi F, Manca M, Leardini A (2012). Estimation of spatial-temporal gait parameters in level walking base on a single accelerometer: validation on normal subjects by standard gait analysis. Comput Methods Programs Biomed.

[21] Mazzà C, Donati M, McCamley J, Picerno P, Cappozzo A (2012). An optimized Kalman filter for the estimation of trunk orientation from inertial sensors data during treadmill walking. Gait Posture.

[22] Bonnet V, Mazzà C, McCamley J, Cappozzo A (2013). Use of weighted Fourier linear combiner filters to estimate lower trunk 3D orientation from gyroscope sensors data. J Neuroeng Rehabil.

[23] Luinge HJ, Veltink PH (2005). Measuring orientation of human body segments using miniature gyroscopes and accelerometers. Med Biol Eng Comput.

[24] Grimpampi E, Bonnet V, Taviani A, Mazzà C (2013). Estimate of lower trunk angles in pathological gaits using gyroscope data. Gait Posture.

[25] Leardini A, Lullini G, Giannini S, Berti L, Ortolani M, Caravaggi P (2014). Validation of the angular measurements of a new inertial-measurement-unit based rehabilitation system: comparison with state-of-the-art gait analysis. J Neuroeng Rehabil.

[26] Menz HB, Lord SR, Fitzpatrick RC (2003). Acceleration patterns of the head and pelvis when walking are associated with risk of falling in community-dwelling older people. J Gerontol.

[27] Ishigaki N, Kimura T, Usui Y, Aoki K, Narita N, Shimizu M, Hara K, Ogihara N, Nakamura K, Kato H, Ohira M, Yokokawa Y, Miyoshi K, Murakami N, Okada S, Nakamura T, Saito N (2011). Analysis of pelvic movement in the elderly during walking using a posture monitoring system equipped with a triaxial accelerometer and gyroscope. J Biomech.

[28] Bolink SAAN, van Laarhoven SN, Lipperts M, Heyligers IC, Grimm B (2012). Inertial sensor motion analysis of gait, sit-stand transfers and step-up transfers: differentiating knee patients from healthy controls. Physiol Meas.

[29] Bonnechère B, Jansen B, Salvia P, Bouzahouene H, Omelina L, Moiseev F, Sholukha V, Cornelis J, Rooze M, Van Sint JS (2014). Validity and reliability of the Kinect within functional assessment activities: comparison with standard stereophotogrammetry. Gait Posture.

[30] Zijlstra W, Hof At L (2003). Assessment of spatial-temporal gait parameters from trunk accelerations during human walking. Gait Posture.

[31] Iosa M, Cereatti A, Cappozzo A (2009). A linear method for curve comparison in gait data. Gait Posture.

[32] Benedetti MG, Merlo A, Leardini A (2013). Inter-laboratory consistency of gait analysis measurements. Gait Posture.

